# Supplementary material for: Profile of TREM2-Derived circRNA and mRNA Variants in the Entorhinal Cortex of Alzheimer’s Disease Patients
Source: Int J Mol Sci. 2022 Jul 12;23(14):7682. doi: 10.3390/ijms23147682 (PMC9320643; doi:10.3390/ijms23147682)
Supplement: Supplementary file 1 [file ijms-23-07682-s001.zip › Additional Figure s1.pdf]

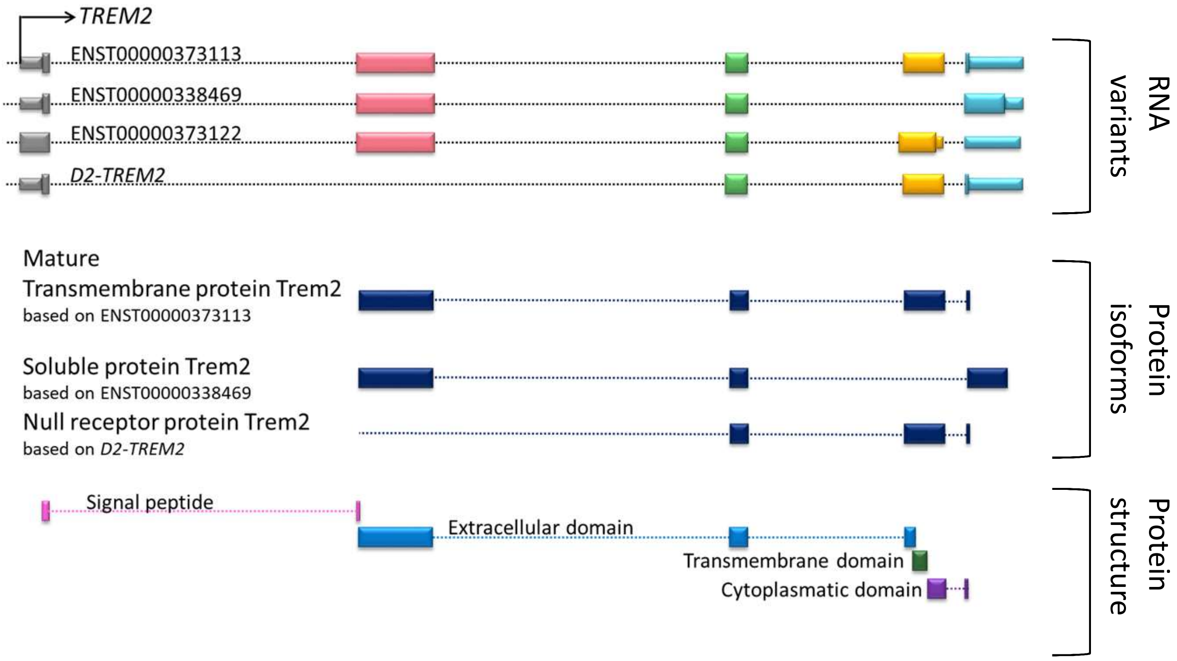

Additional Figure S1: TREM2 RNA and protein maps. The image shows the RNA variants of *TREM2* and 2 of the Trem2 protein isoforms, where the boxes represent the exons (top). In the case of the protein variants, the boxes show the exons that are translated into protein. In the lower part of the figure, the domains of the protein are aligned to the exons.
