# Supplementary material for: Profile of TREM2-Derived circRNA and mRNA Variants in the Entorhinal Cortex of Alzheimer’s Disease Patients
Source: Int J Mol Sci. 2022 Jul 12;23(14):7682. doi: 10.3390/ijms23147682 (PMC9320643; doi:10.3390/ijms23147682)
Supplement: Supplementary file 1 [file ijms-23-07682-s001.zip › Additional Figure s3.pdf]

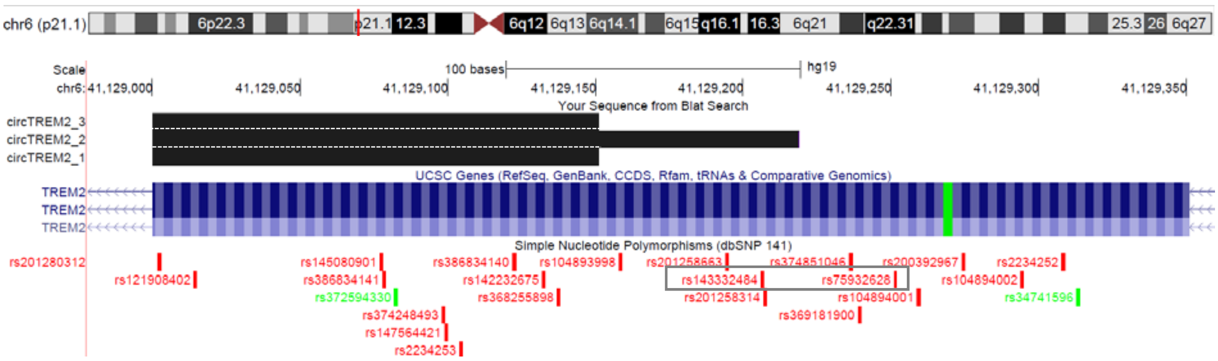

Additional Figure S3. In exon 2 of *TREM2* has been described 2 SNPs that are risk factors of AD. The image shows in black the sequence of exon 2 included in each circTREM2, in blue the exon 2 of the TREM2 gene and in red and green the SNPs described in this exon, the 2 in the grey box are the ones described as risk factors for AD.
