# Supplementary material for: Profile of TREM2-Derived circRNA and mRNA Variants in the Entorhinal Cortex of Alzheimer’s Disease Patients
Source: Int J Mol Sci. 2022 Jul 12;23(14):7682. doi: 10.3390/ijms23147682 (PMC9320643; doi:10.3390/ijms23147682)
Supplement: Supplementary file 1 [file ijms-23-07682-s001.zip › Additional Figure s5.pdf]

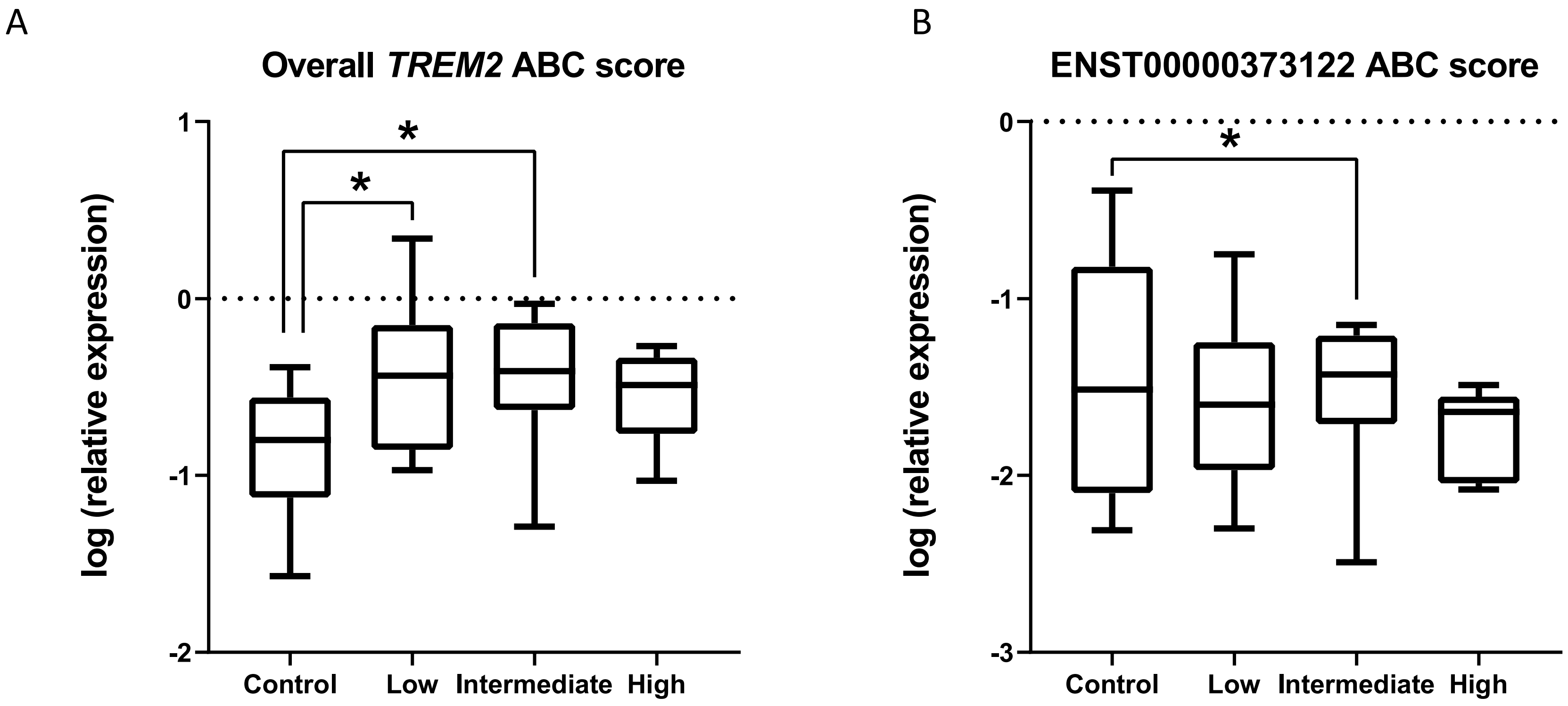

Additional figure S5. Expression of *TREM2* mRNA by ABC score. A) Box-plot represents the expression of *TREM2* mRNA by ABC score. B) Box-plot represents the expression of ENST00000373122 transcript by ABC score. Vertical lines represent the SE.*p-value<0.05.
