# Supplementary material for: Profile of TREM2-Derived circRNA and mRNA Variants in the Entorhinal Cortex of Alzheimer’s Disease Patients
Source: Int J Mol Sci. 2022 Jul 12;23(14):7682. doi: 10.3390/ijms23147682 (PMC9320643; doi:10.3390/ijms23147682)
Supplement: Supplementary file 1 [file ijms-23-07682-s001.zip › Additional table s1.pdf]

Additional Table S1: Sequence of set of primers used for RT-PCR and RTqPCR experiments

Divergent PCR primers set

			Amplicon molecular weight (bp) expected for each circTREM2/primer set		
	**Forward 5'->3'**	**Reverse 5'->3'**	**circTREM2_1**	**circTREM2_2**	**circTREM2_3**
**circTREM2_4-5**	GTGGGGAGGTGGTAAGAACA	CCAGGAGGAGAAGGATGGA	-	-	-
**circTREM2_3-4**	ACAGAAGCCAGGGACACATC	CATCCTCGAAGCTCTCAGAC	358	427	306
**circTREM2_2-4**	ACAGAAGCCAGGGACACATC	CTGGTAGAGACCCGCATCAT	228	297	176
**circTREM2_2-3**	CATGTGGAGCACAGCATCTC	CTGGTAGAGACCCGCATCAT	368	437	316
**circTREM2_3-5**	GAAAAGCCCAGGAGAAGTCC	GGAACCAGAGATCTCCAGCA	221	-	290

TaqMan assay

	**Forward 5'->3'**	**Reverse 5'->3'**	**Probe 5'->3'**
**circTREM2**	CAGGGTATCAGCTCCAAACTC	AGCGTAATGGTGAGAGTGC	ATCGTCTGTGATGGCTGTGCTGG
**ACTB**	ACCTTCTACAATGAGCTGCG	CCTGGATAGCAACGTACATGG	ATCTGGGTCATCTTCTCGCGGTTG

SYBR Green assay

	**Forward 5'->3'**	**Reverse 5'->3'**
***TREM2* _overall**	CTGCTCATCTTACTCTTTGTCAC	CAGTGCTTCATGGAGTCATAGG
**ENST00000373122**	TTCGAGGATGCCCATGTG	GGAGAGACAAGAAGGCAGATG
**ENST00000373113**	GCATCTCCAGGAGCCTCT	ATGTGTCCCTGGCTTCTGTC
**ENST00000338469**	AGCCATCACAGACGATACCC	TCTCAGCCCTGGAGATGC
D2-TREM2*	TTACTCTTTGTCACAGACCCC	GGGCATCCTCGAAGCTCT
GAPDH	ACATCGCTCAGACACCATG	TGTAGTTGAGGTCAATGAAGGG
ACTB	GGACTTCGAGCAAGAGATGG	AGCACTGTGTTGGCGTACAG

*Original reference is provided in 5. Materials and methods section
