# Supplementary material for: Profile of TREM2-Derived circRNA and mRNA Variants in the Entorhinal Cortex of Alzheimer’s Disease Patients
Source: Int J Mol Sci. 2022 Jul 12;23(14):7682. doi: 10.3390/ijms23147682 (PMC9320643; doi:10.3390/ijms23147682)
Supplement: Supplementary file 1 [file ijms-23-07682-s001.zip › Additional table s2.pdf]

Additional Table S2. Brain sample set characteristics. The table shows the characteristic of the samples included in the study. No.: Number; NPD: no protein deposit; h: hours; PMI: *post mortem* interval.

Nº	Braak stage	ABC score	ABC scale	Gender	% Aβ plaque area	Age at death (years)	PMI (h)
1	III	A1B2C1	Low	Female	0,349	96	1,5
2	III	A1B2C1	Low	Female	0,044	88	33
3	I	A1B1C1	Low	Female	0.00	85	4,3
4	NA	NA	NA	Female	0,672	69	NA
5	II	A1B1C1	Low	Female	0,147	66	1,4
6	III	A1B2C3	Int	Female	0,648	84	13
7	IV	A2B2C2	Int	Female	0,814	97	NA
8	IV	A2B2C3	Int	Male	0,420	78	5
9	I	A1B1C1	Low	Male	0,000	60	15,3
10	V	A3B3C2	High	Male	NA	91	5
11	III	A3B2C3	Int	Male	0,724	83	9
12	IV	A3B2C1	Int	Female	0,131	90	3
13	I	A1B1C1	Low	Male	0,024	85	3,2
14	III	A3B2C3	Int	Female	0,376	85	NA
15	III-IV	A3B2C3	Int	Female	0,357	98	3
16	IV	A2B2C2	Int	Female	0,431	91	10
17	III	A2B2C3	Int	Female	0,528	98	23
18	V	A3B3C3	High	Female	0,777	77	11
19	VI	A3B3C3	High	Female	0,777	86	2,3
20	V	A3B3C2	High	Female	1,610	82	9
21	I	A1B1C1	Low	Female	NA	85	2
22	IV	A2B2C2	Int	Male	0,959	88	3,5
23	VI	A3B3C3	High	Male	2,013	70	2,35
24	II	A2B1C3	Low	Male	NA	80	3
25	II	A1B1C1	Low	Male	NA	74	2,5
26	II	A1B2C2	Int	Female	NA	71	4
27	II	A2B1C1	Low	Female	NA	80	3,7
28	0	control	Not	Female	NPD	43	3
29	0	control	Not	Male	NPD	54	18
30	0	control	Not	Female	NPD	19	NA
31	0	control	Not	Female	NPD	46	7
32	0	control	Not	Male	NPD	28	6
33	0	control	Not	Male	NPD	41	3,5
34	0	control	Not	Male	NPD	54	2,7
35	0	control	Not	Male	NPD	81	10,5
36	0	control	Not	Male	NPD	26	6,2
37	0	control	Not	Male	NPD	53	7
38	0	control	Not	Female	NPD	88	9
39	0	control	Not	Male	NPD	66	6,5
40	0	control	Not	Female	NPD	88	3,5
41	0	control	Not	Female	NPD	76	11,5
42	0	control	Not	Male	NPD	65	3
43	0	control	Not	Male	NPD	83	NA
