# Supplementary figures and images for: Profile of TREM2-Derived circRNA and mRNA Variants in the Entorhinal Cortex of Alzheimer’s Disease Patients
Source: Int J Mol Sci. 2022 Jul 12;23(14):7682. doi: 10.3390/ijms23147682 (PMC9320643; doi:10.3390/ijms23147682)

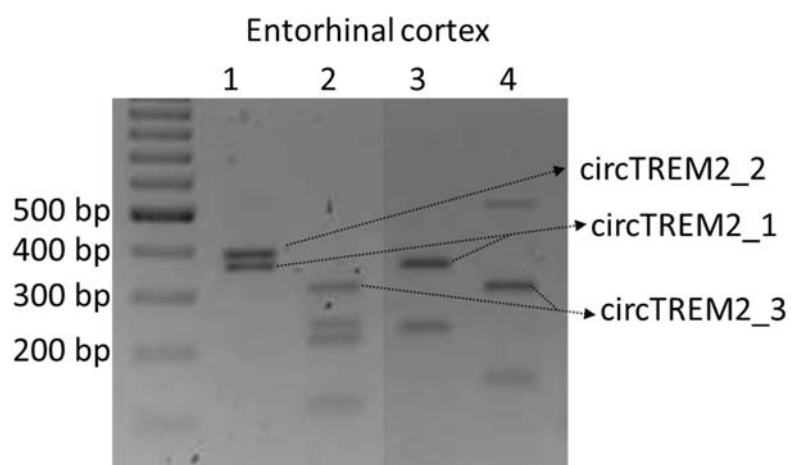

**Additional Figure S2. RT-PCR products of circTREM2\_3-4 primers set in agarose gel.**

Supplement: Supplementary file 1 [file ijms-23-07682-s001.zip › Additional Figure s2.pdf]

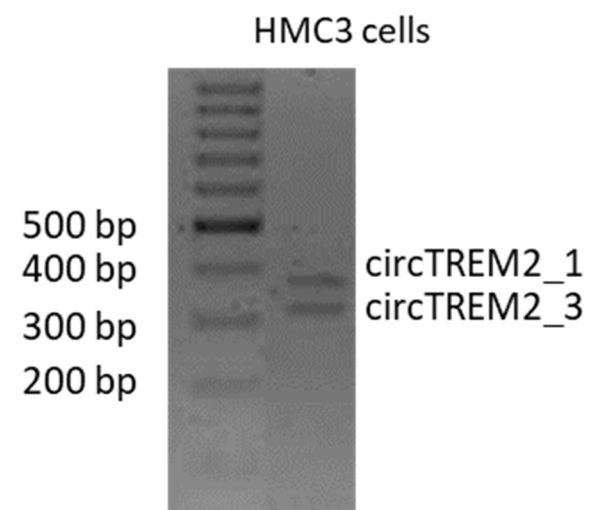

**Additional Figure S4.** RT-PCR products of circTREM2\_2-3 primers set from HMC3 cells in agarose gel.

Supplement: Supplementary file 1 [file ijms-23-07682-s001.zip › Additional Figure s4.pdf]
